# Heterosexual Rejection and Mate Choice: A Sociometer Perspective

**DOI:** 10.3389/fpsyg.2015.01846

**Published:** 2015-12-01

**Authors:** Lin Zhang, Shen Liu, Yue Li, Lu-Jun Ruan

**Affiliations:** ^1^Department and Institute of Psychology, Ningbo UniversityNingbo, China; ^2^Social Cognition and Behavior Laboratory, Ningbo UniversityNingbo, China; ^3^Zhejiang Agricultural Business CollegeShaoxing, China

**Keywords:** heterosexual rejection, mate choice, sociometer theory, self-perceived mate value, mate expectation, mating behavior tendency, self-esteem

## Abstract

Previous studies about the effects of social rejection on individuals' social behaviors have produced mixed results and tend to study mating behaviors from a static point of view. However, mate selection in essence is a dynamic process, and therefore sociometer theory opens up a new perspective for studying mating and its underlying practices. Based on this theory and using self-perceived mate value in the relationship between heterosexual rejection and mate choice as a mediating role, this current study examined the effects of heterosexual rejection on mate choice in two experiments. Results showed that heterosexual rejection significantly reduced self-perceived mate value, expectation, and behavioral tendencies, while heterosexual acceptance indistinctively increased these measures. Self-perceived mate value did not serve as a mediator in the relationship between heterosexual rejection and mate expectation, but it mediated the relationship between heterosexual rejection and mating behavior tendencies toward potential objects. Moreover, individuals evaded both rejection and irrelevant people when suffering from rejection.

## Introduction

The longing for positive and lasting social relations is one of the most pervasive and fundamental human needs (Baumeister and Leary, 1995). Being rejected by social groups is aversive and often threatening. Social rejection refers to all cases that threaten individuals' affinity and need for belonging, which mainly contains being excluded, refused, discriminated against, or neglected by others or groups (Leary et al., 1995).

Rejection elicits negative self-referential cognitions concerning one's social worth and esteem (Monroe et al., 2007; Slavich et al., 2010) and rejected individuals tended to attribute the rejection to theirs or others' shortcomings. They might also blame others (Cheuk and Rosen, 1994) and this attribution often results in a strong feeling of self-frustration (Twenge et al., 2002). Social rejection is related to individuals' negative emotions or feelings and it induces even more negative emotions (Blackhart et al., 2009).

The study on the effects of social rejection on individuals' social behaviors has produced mixed results. Some studies showed that individuals maintain a high willingness of social interaction even when being rejected in order to meet their belonging needs (Williams and Sommer, 1997; Williams et al., 2000; Maner et al., 2007). It is reasonable for the rejected individuals to rebuild relationships to make up for their damaged needs and relationships (Thau et al., 2007). On the flipside, other studies showed that social rejection not only decreases the willingness of social interaction, but also causes individuals to evade rejection and irrelevant people (Vangelisti et al., 2005). Moreover, rejection resulted in a decrease of pro-social behaviors (Twenge et al., 2007) and could even arouse aggressive behaviors (Twenge et al., 2001).

The current study took heterosexual rejection as a special form of social rejection, which means rejection and exclusion from the opposite sex, especially potential lovers or spouses. From the perspective of evolutionary psychology, sexual relations are particularly important to human beings due to the fact that they are among the main sources for individuals to achieve belonging, survival, and reproduction. Heterosexual rejection is a common social phenomenon (Pass et al., 2010) and may cause strong physiological and psychological reactions, such as accelerated secretion of salivary cortisol (Ford and Collins, 2010). Compared with refusals from friends, heterosexual rejection causes a significant decline in self-esteem (Pass et al., 2010) and gives rise to suicide, killings (Barber, 2011), and impulsive shopping (Griskevicius et al., 2011; Sundie et al., 2011). However, very little is known about how heterosexual rejection influences one's mating choice. A fundamental question is whether heterosexual rejection increases more uncertainty for mate choice than same-sex rejection? And if so, why? This question has been underappreciated to some degree because early theories could not postulate powerful explanations.

Mate selection has been a hot topic and gained much attention from diverse fields. Some mature theoretical systems have been gradually established, such as the complementary needs theory, the social interaction theory, and so on. However, most of them are based on a static point of view and unable to explain the dynamic process of individuals' mating behaviors. For example, according to social interaction theory, mate choice is in essence a process of give and take. Males and females achieve maximum mutual exchange from each other depending on their own resources (Alterovitz and Mendelsohn, 2009). Mate selection is not only a static phenomenon, such as in terms of mate preferences, but also a dynamic process. People are often inclined to seek out a perfect spouse, but high mate expectations may lead to refusal easily. Moreover, how do feedbacks from others during mate selection influence one's social actions in the future? Therefore, it is not enough to study individuals' mating behaviors only from a static view. The sociometer theory proposed by Mark Leary and his colleagues opens up a new perspective for this concept and its underlying dynamic mate process (Leary and Downs, 1995; Leary et al., 1995; Leary and Baumeister, 2000). Leary et al. mainly answered two fundamental questions: (a) what exactly is self-esteem? and (b) what is its function (Kavanagh et al., 2010)? They found that self-esteem is not a free-floating goal state that people are motivated to enhance and protect. Rather, it is an internal index or gauge—a sociometer—designed to monitor success with respect to other adaptive goals. Self-esteem is considered as a barometer of one's perceived past, present, and future relational value (Anthony et al., 2007), an inner reflection of the quality of individual relationships (Zhang and Li, 2009), and it represents the extent of acceptance from others (Leary et al., 1995). Self-esteem declines in face of rejection (Blackhart et al., 2009) and causes negative emotional reactions. In order to maintain the original self-evaluation, individuals take actions to restore good relationships (Zhang and Cao, 2011). Therefore, the sociometer works as a psychological mechanism that monitors individuals' social environments and reflects the degree of one's social inclusion or acceptance vs. social exclusion or rejection, and its high reliability and effectiveness have been demonstrated by numerous studies (Back et al., 2009; Li, 2014).

Kirkpatrick and Ellis (2001) proposed an extension of sociometer theory that there are multiple sociometers with multiple functions associated with functionally distinct social-psychological systems, and one of them is the function of guiding adaptive relationship choices. In the context of developing relationships in different social domains, individuals face the problem of adaptively calibrating their levels of aspiration. Kirkpatrick and Ellis (2001) have hypothesized that an important function of self-esteem is to guide individuals to build relatively high-quality yet defensible social relationships, given one's own social value. Their model posits that the experiences of social acceptance and rejection feed into domain-specific sociometers, causing alterations in state self-esteem in relevant social domains, which, in turn, affecting aspiration levels in approaching new relationships in that domain. The mating sociometer is an important part of human mating intelligence and it is the application of specific sociometer in the field of mating (Kirkpatrick and Ellis, 2001; Greengross and Miller, 2011). From the perspective of evolutionary psychology, the human race faces two major problems: survival and reproduction, which means individuals need to actively integrate into the society and find ideal spouses for reproduction purposes. In order to maintain long-term intimate relationships and to avoid relationship breakdowns, individuals try their best to gain affirmation and acceptance from others (Baumeister and Leary, 1995; Barber, 2011; Zhang and Cao, 2011). Armed with such knowledge, individuals frequently make a series of adjustments in order to avoid future rejection (Molden et al., 2009; Griskevicius et al., 2012), such as ingratiating themselves (Romero-Canyas et al., 2010), spending money to fit in (Mead et al., 2011) and so on. Others might lower their mate choice criteria or build long-term relationships (Edlund and Sagarin, 2014). During this process, the mating sociometer plays a fatal role. According to Kirkpatrick and Ellis (2001), self-esteem is an inner psychological reflection in mating and alliance. Moreover, the controlled range of this sociometer is interpersonal relationships. In the field of mating, namely the self-perceived mate value, self-esteem monitors the quality of the opposite sex relationships, regulates the level of effort put into mating, and inspires individuals to take actions to maintain or restore the status of being accepted by the opposite sex. Given the findings of previous studies, we postulate that self-perceived mate value plays an important role in humans' preferences of mate selection. For instance, men's and women's demands of mates differ based on their own mate values (Edlund and Sagarin, 2010), and men with lower mate value may resort to cost-inflicting mate retention behaviors that rely on manipulation, intimidation, and possessiveness (Starratt and Shackelford, 2012).

The purpose of this study is to examine the influence of heterosexual feedback on mate choice. Scene-priming and speed-date paradigms were adopted to investigate the effect of immediate heterosexual rejection on mate value and the function of self-perceived mate value during this process. Based on those aforementioned researches, we propose our hypotheses as follows: (1) Heterosexual rejection reduces self-esteem and self-perceived mate-value, and the reducing effect on the latter is stronger than that on the former, (2) Heterosexual rejection reduces individuals' mate expectations in which self-perceived mate value may serve as a mediator, and (3) Heterosexual rejection causes individuals to evade rather than approach and self-perceived mate value may mediate the relationship between heterosexual rejection and mating behavior tendencies.

## Experiment 1

### Methods

#### Participants and design

One hundred and fifty Chinese undergraduate students, aged between 20 and 30 years (75 males: *M* = 23.54, *SD* = 1.83; 75 females: *M* = 22.62, *SD* = 1.74) were recruited, and they all reported as heterosexual. Each participant gave written informed consent to take part in the study and received a payment of 10 Yuan (RMB) as reward. This study is also approved by the Human Research Ethics Committee of Ningbo University.

### Materials

#### Scene-priming

Three scene-priming materials were compiled by adapting different standards to fit into college students' lifestyle, scene description processes, and similar contents, and were then modified by several experts. Thirty college students participated in the preliminary experiment, with 10 participants testing each type of material with the dependent variable being social acceptance. Only female participants were selected to test the effectiveness of the materials. The results revealed that there were significant differences in the three types of materials when participants were only exposed to scenes (*p* < 0.05), e.g., the scene is in a big party, both males and females could invite their favorite heterosexual partners to dance with them. When Lily invited a male, she was rejected. Please answer the following questions: (1) If you were Lily, please describe briefly what did you think when being rejected? (2) Please think carefully whether you have experienced similar rejection by the opposite sex and recall it as vividly as possible. (3) Describe the process of being rejected, your feelings in that moment, and its subsequent influences. All the three scenarios were presented in the Supplementary Material.

#### Social acceptance

According to Kavanagh et al. (2010), four paired groups of adjectives were chosen to measure social acceptance: liked-disliked, popular-unpopular, socially attractive-socially unattractive, and accepted-rejected. A scale ranging from 1 (*strongly disagree*) to 7 (*strongly agree*) was used to measure participants' feelings when experience rejection or acceptance. In this study, Cronbach's alpha coefficient was 0.92.

#### State self-esteem

According to Heatherton and Polivy (1991), 12 paired groups of adjectives were chosen to measure state self-esteem: fine-awful, competent-incompetent, proud-ashamed, fit-unfit, useful-useless, excellent-bad, smart-stupid, confident-self-abased, valuable-worthless, important-negligible, efficient-inefficient, and satisfied-unsatisfied. A scale ranging from 1 (*strongly disagree*) to 7 (*strongly agree*) was used to measure participants' state self-esteem. In this study, Cronbach's alpha coefficient was 0.92.

#### Self-perceived mate value

Self-perceived mate value was assessed using the Self-Perceived Mating Success Scale (Landolt et al., 1995), which contains 10 items measuring the extent to which individuals believed they can attract mates of the opposite sex. Participants rated their agreement on the same 7-point scale as above. In this study, Cronbach's alpha coefficient was 0.93.

All the materials used in this experiment were presented in Chinese, and several English native speakers were invited to help translating these materials.

### Procedure

Firstly, all participants were randomly divided into three groups with an equal number of males and females. Then, participants in different groups were presented with different scene materials: heterosexual rejection, same-sex rejection, and heterosexual acceptance. Instructions were given to scan materials and answer questions attentively. Upon finishing, participants completed all the scales used in the study. Finally, participants were debriefed with regard to the rationale and deceptions of this study, given instructions not to discuss the contents of the study with anyone, and then thanked and dismissed.

### Results

#### Manipulation checks

To investigate the effects of manipulation, a single factor (types of rejection) between subject MANOVA was conducted. Social acceptance was adopted as an index of manipulation checks. The results indicated that there were significant differences in social acceptance of different experimental manipulations, *F*_(2, 147)_ = 9.61, *p* < 0.001, $\eta_{p}^{2}=0.12$. An LSD test was used to further clarify the differences among groups and showed that social acceptance of the heterosexual rejection group (*t* = −3.76, *p* < 0.01, *d* = −0.97) and the same-sex rejection group (*t* = −2.99, *p* < 0.01, *d* = −0.64) were significantly lower than that of the heterosexual acceptance group, while there was no significant difference between the heterosexual rejection group and the same-sex rejection group (*t* = −0.77, *p* = 0.399, *d* = −0.15).

#### Self-perceived mate value and self-esteem of different rejected groups

According to the sociometer theory, social rejection has an effect on individuals' self-esteem (Leary et al., 1995). First of all, we investigated the domain difference between self-perceived mate value and self-esteem.

To investigate the domain difference between self-perceived mate value and self-esteem, a single factor (types of rejection) between subject MANOVA was conducted. As illustrated in Table 1, there were significant differences between the groups of self-perceived mate value [*F*_(2, 147)_ = 8.67, *p* < 0.001, $\eta_{p}^{2}=0.11$] and state self-esteem [*F*_(2, 147)_ = 7.35, *p* < 0.001, $\eta_{p}^{2}=0.09$]. An LSD test was used to further clarify the differences among groups and showed that self-perceived mate value of the heterosexual rejection group was significantly lower than that of the same-sex rejection group (*t* = −3.86, *p* < 0.01, *d* = −0.56) and the heterosexual acceptance group (*t* = −5.60, *p* < 0.001, *d* = −0.76), while no significant difference was found between the same-sex rejection group and the heterosexual acceptance group (*t* = −1.74, *p* = 0.072, *d* = -0.28). State self-esteem of the heterosexual rejection group (*t* = −9.74, *p* < 0.001, *d* = −0.72) and the same-sex rejection group (*t* = −8.37, *p* < 0.01, *d* = −0.63) were significantly lower than the heterosexual acceptance group, while there was no significant difference between the heterosexual rejection group and the same-sex rejection group (*t* = −1.37, *p* = 0.062, *d* = 0.09). These findings indicated that self-perceived mate value was more sensitive to heterosexual rejection than self-esteem. The results above showed that rejection did influence one's state self-esteem. To eliminate the effect of state self-esteem on self-perceived mate value, we investigated the effect of heterosexual rejection on self-perceived mate value by using state self-perceived mate value as a covariate. The result showed that the types of rejection had a significant effect on self-perceived mate value when state self-esteem was controlled, *F*_(2, 146)_ = 5.17, *p* < 0.01, $\eta_{p}^{2}=0.07$.

**Table 1:** **The difference between self-perceived mate value and self-esteem in Experiment 1 (*M* ± *SD*)**.

**Types of rejection**	**Self-perceived mate value**	**State self-esteem**
Heterosexual rejection (*n* = 50)	35.96 ± 7.80	49.85 ± 14.72
Same-sex rejection (*n* = 50)	39.82 ± 5.70	51.22 ± 14.45
Heterosexual acceptance group (*n* = 50)	41.56 ± 7.00	59.59 ± 11.91
*F*	8.67^***^	7.35^**^

** p < 0.01,

*** p < 0.001.

#### Effects of gender and relationship status on self-perceived mate value of different rejection types

Effects of gender and relationship status on self-perceived mate value of different rejection types are illustrated in Table 2.

**Table 2:** **Effect of gender on self-perceived mate value of different rejection types in Experiment 1 (*M* ± *SD*)**.

**Types of rejection**	**Male**	**Female**
Heterosexual rejection (*n* = 50)	34.35 ± 8.90	37.70 ± 6.10
Same-sex rejection (*n* = 50)	39.74 ± 4.58	39.89 ± 6.58
Heterosexual acceptance group (*n* = 50)	41.57 ± 7.14	41.55 ± 6.99

To further clarify the effects of heterosexual and same-sex rejection on self-perceived mate value, a 2 (gender) × 3 (types of rejection) MANOVA was conducted. It revealed a significant effect of experimental manipulations: *F*_(2, 144)_ = 8.38, *p* < 0.001, $\eta_{p}^{2}=0.10$. The LSD test showed that self-perceived mate value of the heterosexual rejection group was significantly lower than that of the same-sex rejection group (*t* = −3.79, *p* < 0.01, *d* = −3.89) and the heterosexual acceptance group (*t* = −5.53, *p* < 0.001, *d* = −5.67), while no significant difference was reported between the same-sex rejection group and the heterosexual acceptance group (*t* = −1.74, *p* = 0.210, *d* = 1.78). The main effect of gender [*F*_(1, 144)_ = 1.06, *p* = 0.306, $\eta_{p}^{2}=0.01$] and the interaction between gender and experimental manipulations [*F*_(2, 144)_ = 0.95, *p* = 0.389, $\eta_{p}^{2}=0.01$] reported no significant difference.

Considering the effect of relationship status, we then conducted a 3 (types of rejection) × 2 (relationship status) MANOVA. The results revealed a significant effect of experimental manipulations [*F*_(2, 144)_ = 9.95, *p* < 0.001, $\eta_{p}^{2}=0.13$]. The LSD test showed that self-perceived mate value of the heterosexual rejection group was significantly lower than that of the same-sex rejection group (*t* = −4.85, *p* < 0.01, *d* = −4.45) and the heterosexual acceptance group (*t* = −6.13, *p* < 0.001, *d* = −0.61), while no significant difference was reported between the same-sex rejection group and the heterosexual acceptance group (*t* = −1.28, *p* = 0.422, *d* = −0.82). The main effect of relationship status reported significant differences [*F*_(1, 144)_= 8.20, *p* < 0.01, $\eta_{p}^{2}=0.06$]. The LSD test showed that self-perceived mate value of the single was significantly lower than that of the double (*t* = −3.58, *p* < 0.01, *d* = −4.10). The interaction between experimental manipulations and relationship status reported no significant difference [*F*_(2, 144)_ = 0.11, *p* = 0.895, $\eta_{p}^{2}=0.01$].

### Discussion

The current study found that different types of rejection cause a change in participants' self-perceived mate value. To be specific, heterosexual rejection significantly lowered one's self-perceived mate value, while same-sex rejection did not. It was clear that rejection itself did not influence one's self-perceived mate value but rejection from the opposite sex did. An analysis of gender difference revealed that the self-perceived mate value of males significantly reduced when they suffered from heterosexual rejection while females reported no significant difference. According to evolutionary psychology, self-perceived mate value of different genders is determined by different factors. Generally speaking, self-perceived mate value of males is more related to competence and social status, while that of females is external attractiveness (Li et al., 2002). We also found that individuals in different relationship status showed significant differences in self-perceived mate value. Compared with those who were single, individuals in love exhibited more enthusiasm in participating activities (Whitty, 2015). In this way, more opportunities to get along with the opposite sex increases self-perceived mate value. Moreover, if individuals in love gain positive feedbacks from the opposite sex, this would in turn induce more positive self-evaluation. The two reasons above seem to influence the difference in self-perceived mate value of individuals in different relationship status. Subsequent research should control relationship status to avoid the results being skewed.

Self-esteem can be divided into general self-esteem and specific self-esteem, and the latter is also called specific self-evaluation, which primarily considers individuals' evaluation and judgment of all of their competencies and qualities (Zhang and Li, 2009). For example, one who considers himself of high academic ability is regarded as having high academic self-esteem; one who considers himself of low kinesthetic ability is probably thought to possess low athletic self-esteem. In view of the above, it can be inferred that individuals have different self-esteems in different domains. Individuals with high athletic self-esteem may have low artistic self-esteem and individuals with low academic self-esteem may have high sociable self-esteem. According to sociometer theory in specific domain, psychological mechanisms in different domains have their corresponding sociometers to monitor. In the domain of mate selection, self-esteem performed as self-perceived mate value, which is individuals' judgment of their own competencies and qualities in mating. According to the sociometer theory, self-esteem is thought to be an inner reflection of the quality of interpersonal relations. Self-evaluation is influenced by social feedback and social rejection significantly lowers state self-esteem (Leary et al., 1995). Although the current results showed that rejection did influence one's self-esteem, different types of rejection reported no significant difference in effect. Heterosexual and same-sex rejection are equivalent to one's overall self-esteem, meaning that the overall self-esteem is a sociometer of domain generality. In contrast, self-perceived mate value as specific self-esteem reacts differently when exposed to heterosexual and same-sex rejection. Heterosexual rejection significantly influences self-perceived mate value while same-sex rejection does not. When controlling overall self-esteem as a covariate, heterosexual rejection still influenced self-perceived mate value, indicating that heterosexual rejection did influence self-perceived mate value in the absence of overall self-esteem.

Experiment 1 preliminarily proved that heterosexual rejection influenced self-perceived mate value and clarified the difference between general self-esteem and specific self-esteem. Based on these results, Experiment 2 further investigated how heterosexual rejection influences mate choice as well as the possible effect of self-perceived mate value during this process.

## Experiment 2

### Methods

#### Participants and design

Sixty Chinese undergraduate students, aged between 18 and 26 years (30 males: *M* = 20.73, *SD* = 1.28; 30 females: *M* = 19.96, *SD* = 1.03) participated for monetary compensation. Those participants were evenly divided into three groups: the rejected group, the accepted group, and the control group. They all reported being heterosexual and single. Written informed consent was obtained before the study. The study is also approved by the Human Research Ethics Committee of Ningbo University.

### Materials

#### Social acceptance

According to Kavanagh et al. (2010), four paired groups of adjectives were chosen to measure social acceptance: liked-disliked, popular-unpopular, socially attractive-socially unattractive, and accepted-rejected. A scale ranging from 1 (*strongly disagree*) to 7 (*strongly agree*) was used to measure participants' feelings when experience rejection or acceptance. The measurement of social acceptance in this experiment was the same as Experiment 1. In this study, Cronbach's alpha coefficient was 0.95.

#### Self-perceived mate value

Self-perceived mate value was assessed using the Self-Perceived Mating Success Scale (Landolt et al., 1995), which contains 10 items measuring the extent to which individuals believed they can attract mates of the opposite sex. Participants rated their agreement on the same 7-point scale as above. The measurement of self-perceived mate value in this experiment was the same as Experiment 1. In this study, Cronbach's alpha coefficient was 0.90.

#### Mate expectation

Firstly, participants selected the opposite sex with the highest scores as their objectives. Then, they answered five questions, such as “Does he/she belong to the category of person you would successfully date?” “How likely do you think this person is interested in you?” Each question was answered on a 5-point scale ranging from 1 (*extremely impossible*) to 5 (*extremely possible*). Higher scores indicate higher mate expectation (Kavanagh et al., 2010). In this study, Cronbach's alpha coefficient was 0.87.

#### Mating behavior tendency

According to Ha et al. (2010), we adopted dating desire as a variable to measure participants' mating behavior tendencies. It consisted of five items which are divided into two indicators. One indicator was present participant, which is applicable for participants who evade individuals present that interacted with them. For example, Jason rejected Anna's invitation in a large party, we referred Jason as a “present participant.” The other indicator was absent participant, which is applicable for participants who evade individuals irrelevant that they might potentially get in touch with in the future. For example, Jason rejected Anna's invitation in a big party. Several days later, John and Anna would attend another party, we considered Jason as an “absent participant.” For both present and absent participants, we asked questions pertaining to their interests in further knowing their opposite-sex participants. Each question was answered on a 5-point scale ranging from 1 (*extremely unwilling*) to 5 (*extremely willing*). It was mainly used to measure participants' dating desires after experiencing different types of feedbacks. In this study, Cronbach's alpha coefficient was 0.88.

All the materials used in this experiment were presented in Chinese, and several English native speakers were invited to help translating these materials.

### Procedure

Participants, evenly divided between males and females, were asked to enter the lab at the same period of time. Instructions were as follows: This is a psychological experiment about how individuals interact with the opposite sex. We want to explore what kinds of impressions would be formed during the process of dating with the opposite sex. Furthermore, we wonder how these impressions would change in further interactions. This activity consisted of both discussions and questionnaire testing, which will take about 30 min. Then, the experimenter numbered each participant. Female participants were instructed to circle in accordance with their number and sit outward. Comparatively, male participants sat in a circle facing female participants according to their number. Each pair of participants was given 3 min to communicate.

The purpose of this activity was to lead participants to form an initial impression of the opposite sex on appearance, personality, language expression, logical thinking, and so on. In this way, they could make evaluations of the opposite sex when the activity ends. Both genders rotated their positions after they chatted with each member of the opposite sex. Then, we arranged everybody to sit in the same row in order to ensure that they could not see each other's questionnaires. After participants scored the opposite sex present and chose one who they were most willing to date, the experimenter pretended to calculate the results. Participants then were told about the results. Feedback manipulations were as follows: For the rejected group, the experimenter told them that they were selected by only one of the opposite sex for further communication, while for the accepted group, they were selected by seven of the opposite sex for more contact. And for the control group, they were told that follow-up questionnaires need to be filled in due to technical errors in counting the results. After this activity, participants were asked to complete the questionnaires used in the experiment and were debriefed the same way as Experiment 1.

### Results

#### Manipulation checks

To investigate the effects of manipulation, a single factor (types of rejection) between subject MANOVA was conducted. Social acceptance was adopted as an index of manipulation checks. The results indicated that there were significant differences in social acceptance of different experimental manipulations, *F*_(2, 57)_ = 35.65, *p* < 0.001, $\eta_{p}^{2}=0.56$. An LSD test was used to further clarify the differences among the groups and showed that social acceptance of the rejected group was significantly lower than that of the accepted group (*t* = −8.73, *p* < 0.001, *d* = −2.52) and the control group (*t* = −5.41, *p* < 0.001, *d* = −1.65), meanwhile social acceptance of the control group was significantly lower than that of the accepted group (*t* = −3.32, *p* < 0.01, *d* = −0.93).

We divided the results into two parts to investigate how heterosexual rejection influences mate expectation and mating behavior tendencies through self-perceived mate values, respectively.

#### Self-perceived mate value as mediator of the relationship between heterosexual rejection and mate expectation

**Figure 1:**
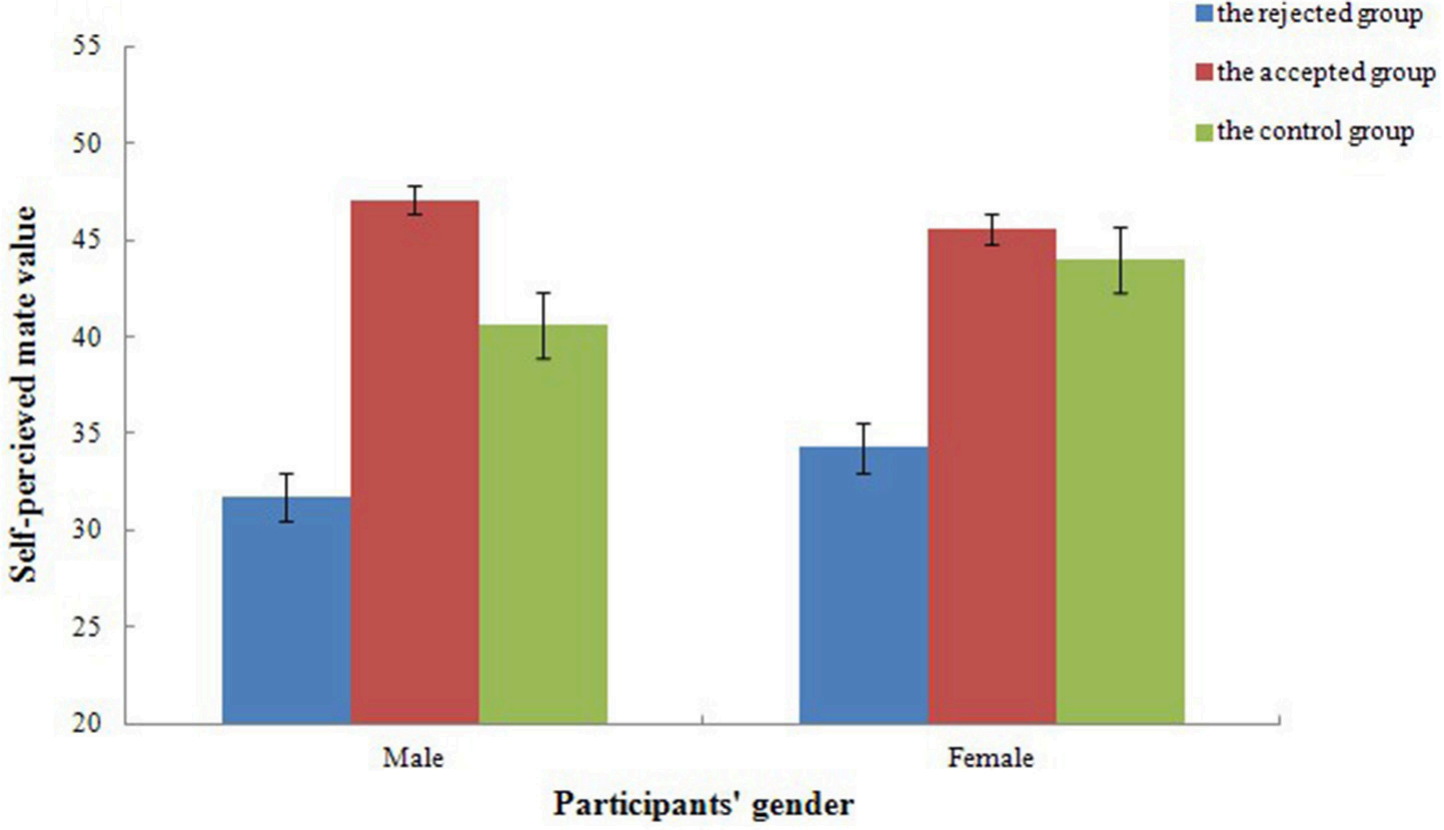
**Self-perceived mate value of both the control group and the accepted feedback group were higher than that of the rejected group in different experimental treatment**. Error bars represent 1 SEM (Standard Error of Mean).

For self-perceived mate value, a mixed repeated measures ANOVAs, 2 (gender) × 3 (experimental treatment), revealed a significant effect of experimental treatment [*F*_(2, 54)_ = 25.57, *p* < 0.001, $\eta_{p}^{2}=0.49$]. As illustrated in Figure 1, self-perceived mate value of the control group (*t* = 9.31, *p* < 0.001, *d* = 6.32) and the accepted group (*t* = 13.32, *p* < 0.001, *d* = 9.91) were higher than that of the rejected group. The main effect of gender [*F*_(1, 54)_ = 0.78, *p* = 0.380, $\eta_{p}^{2}=0.01$] and the interaction between gender and experimental treatment [*F*_(2, 54)_ = 0.88, *p* = 0.419, $\eta_{p}^{2}=0.03$] reported no significant difference.

**Figure 2:**
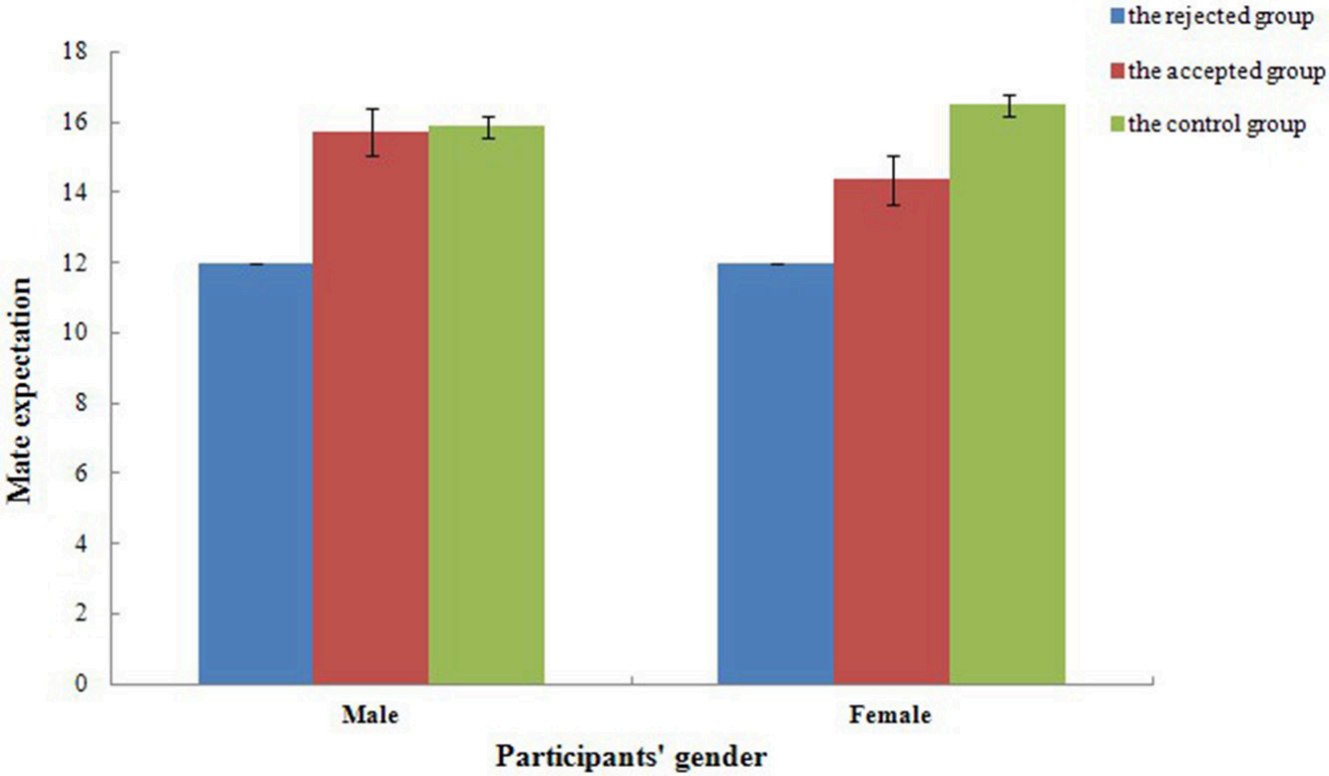
**Mate expectations of both the control group and the accepted feedback group were higher than that of the rejected group in different experimental treatment**. Error bars represent 1 SEM (Standard Error of Mean).

For mate expectation, a mixed repeated measures ANOVAs, 2 (gender) × 3 (experimental treatment), revealed a significant effect of experimental treatment [*F*_(2, 54)_ = 11.76, *p* < 0.001, $\eta_{p}^{2}=0.30$]. As illustrated in Figure 2, mate expectation in the control group (*t* = −4.19, *p* < 0.001, *d* = 6.39) and the accepted group (*t* = −3.05, *p* < 0.001, *d* = 5.09) were higher than that of the rejected group while no significant difference was found between the control group and the accepted group (*t* = 1.14, *p* = 0.221, *d* = 1.74). The main effect of gender [*F*_(1, 54)_ = 0.11, *p* = 0.738, $\eta_{p}^{2}=0.01$] and the interaction between gender and experimental treatment [*F*_(2, 54)_ = 0.64, *p* = 0.530, $\eta_{p}^{2}=0.02$] reported no significant difference. Therefore, participants' self-perceived mate value and mate expectation were not affected by gender, though they might have lowered when participants experience refusals from the opposite sex.

The mediation model of self-perceived mate value did not fit satisfactorily to the data, with χ^2^/df = 5.57, RMSEA = 0.06, GFI = 0.48, NFI = 0.47, and TLI = 0.46. The SEM results revealed a significant direct effect of heterosexual rejection on self-perceived mate value (β = −1.12, *p* < 0.001), while there was not a direct effect of heterosexual rejection on mate expectation (β = −0.17, *p* = 0.097). The SEM results also showed that there was not a significant effect self-perceived mate value on mate expectation (β = 0.09, *p* = 0.155). In addition, bootstrapping showed that there was not an indirect pathway from heterosexual rejection to mate expectation via self-perceived mate value (indirect way: β = −0.26, *p* = 0.109, 95%, CI = −0.37 to 0.04). Therefore, these results indicated that the self-perceived mate value did not mediate the relationship between heterosexual rejection and mate expectation.

#### Self-perceived mate value as mediator of the relationship between heterosexual rejection and mating behavior tendency

**Figure 3:**
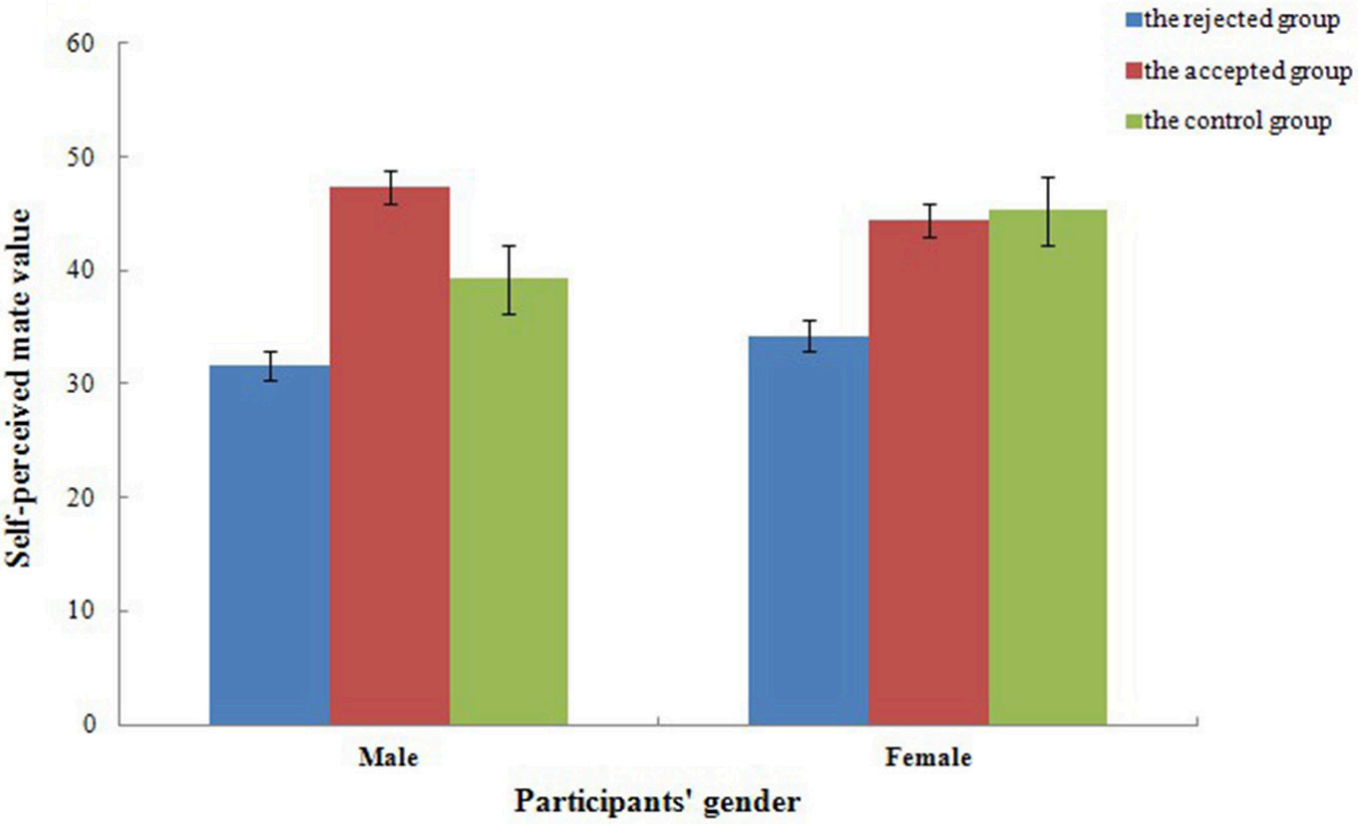
**Self-perceived mate value of the control group and the accepted feedback group were higher than that of the rejected group in different experimental treatment**. Error bars represent 1 SEM (Standard Error of Mean).

For one's self-perceived mate value, a mixed repeated measures ANOVAs, 2 (gender) × 3 (experimental treatment), revealed a significant effect of experimental treatment, *F*_(2, 54)_ = 21.13, *p* < 0.001, $\eta_{p}^{2}=0.44$. As illustrated in Figure 3, self-perceived mate value of the control group (*t* = −9.33, *p* < 0.001, *d* = 6.40) and the accepted group (*t* = −12.98, *p* < 0.001, *d* = 8.93) were higher than that of the rejected group, while no significant difference was reported between the control group and the accepted group (*t* = −3.65, *p* = 0.082, *d* = 2.51). Both the main effect of gender [*F*_(1, 54)_ = 1.31, *p* = 0.258, $\eta_{p}^{2}=0.02$] and the interaction between gender and experimental treatment [*F*_(2, 54)_ = 2.41, *p* = 0.099, $\eta_{p}^{2}=0.08$] reported no significant difference.

**Figure 4:**
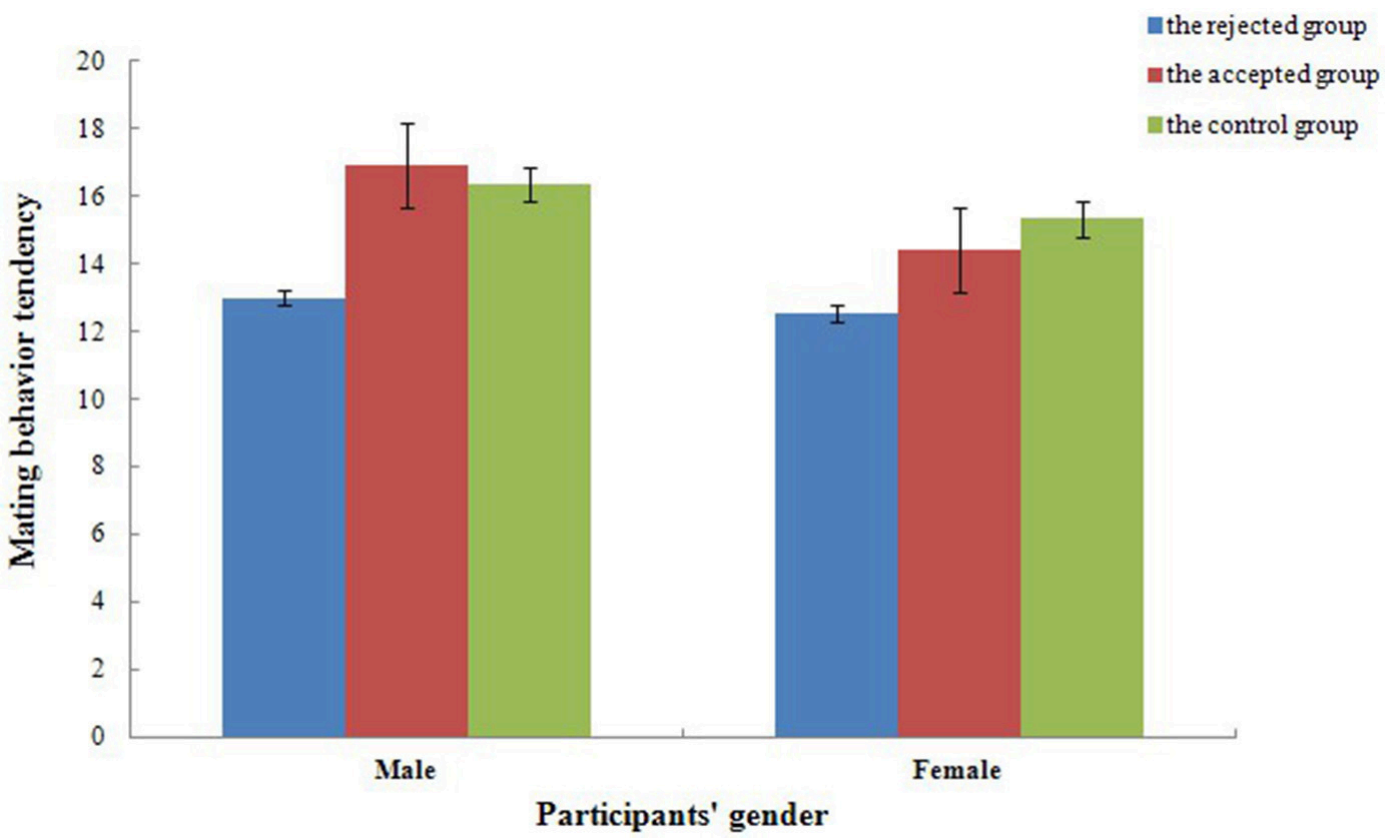
**Mate behavior tendency of the control group and the accepted feedback group were higher than that of the rejected group in different experimental treatment**. Error bars represent 1 SEM (Standard Error of Mean).

For mating behavior tendencies, a mixed repeated measures ANOVAs, 2 (gender) × 3 (experimental treatment), revealed a significant effect of experimental treatment [*F*_(2, 54)_ = 7.88, *p* < 0.001, $\eta_{p}^{2}=0.23$]. As illustrated in Figure 4, the mating behavior tendencies of the control group (*t* = −3.08, *p* < 0.001, *d* = 5.01) and the accepted group (*t* = −2.88, *p* < 0.01, *d* = 4.70) were higher than that of the rejected group while no significant difference was reported between the control group and the accepted group (*t* = 0.20, *p* = 0.820, *d* = −0.32). The main effect of gender [*F*_(1, 54)_ = 3.53, *p* = 0.066, $\eta_{p}^{2}=0.06$] and the interaction between gender and experimental treatment [*F*_(2, 54)_ = 0.74, *p* = 0.480, $\eta_{p}^{2}=0.03$] reported no significant difference. Therefore, participants' mating behavior tendencies were not affected by gender, though it might be lowered when participants face refusals from the opposite sex.

Next, we analyzed the mediating role of self-perceived mate value between heterosexual rejection and mating behavior tendency. The correlation test revealed a positive correlation between one's self-perceived mate value and mating behavior tendency (*r* = 0.27, *p* < 0.05). Following the same procedures above, the mediation model of self-perceived mate value demonstrated a satisfactory fit to the data, with χ^2^/df = 3.28, RMSEA = 0.04, GFI = 0.92, NFI = 0.97, and TLI = 0.96. The SEM results revealed a significant direct effect of heterosexual rejection on mate expectation (β = −0.22, *p* < 0.05) and self-perceived mate value (β = −1.12, *p* < 0.001), but did not show a significant effect self-perceived mate value on mate expectation (β = 0.01, *p* = 0.848). Bootstrapping also showed that there was not an indirect pathway from heterosexual rejection to mating behavior tendencies via self-perceived mate value (indirect way: β = 0.03, *p* = 0.851, 95%, CI = −0.11 to 0.13). Therefore, self-perceived mate value did not work as a mediator for the relationship between heterosexual rejection and mating behavior tendencies.

Furthermore, the mediating role of one's self-perceived mate value between present and absent participants' heterosexual rejection and mating behavior tendencies was investigated. The correlation test did not reveal a positive correlation between self-perceived mate value and present participants' mating behavior tendencies (*r* = 0.05, *p* = 0.076), but a positive correlation was found between that and absent participants' mating behavior tendencies (*r* = 0.28, *p* < 0.05). So, we focused on the effect of heterosexual rejection and self-perceived mate value on absent participants' mating behavior tendencies. The mediation model of self-perceived mate value demonstrated a satisfactory fit to the data, with χ^2^/df = 2.57, RMSEA = 0.02, GFI = 0.98, NFI = 0.97, and TLI = 0.96. The SEM results revealed a significant direct effect of heterosexual rejection on both absent participants' mating behavior tendencies (β = −0.36, *p* < 0.01) and on self-perceived mate value (β = −0.67, *p* < 0.001), and a significant effect of self-perceived mate value on absent participants' mating behavior tendencies (β = 0.28, *p* < 0.05) was found. The total effect of heterosexual rejection on absent participants' mating behavior tendencies experienced a significant drop after self-perceived mate value was controlled (β = −0.32, *p* < 0.05), which meant a significant indirect effect of heterosexual rejection. The Sobel test indicated that an inclusion of self-perceived mate value in the model significant reduced the association between heterosexual rejection and absent participants' mating behavior tendencies, *z* = 2.87, *p* < 0.01. Bootstrapping also showed that an indirect pathway from heterosexual rejection to absent participants' mating behavior tendencies via self-perceived mate value (indirect pathway: β = −0.32, *p* < 0.05, 95%, CI = −0.33 to −0.03). Thus, with respect to the guidelines on the demonstration of mediation (Preacher and Hayes, 2008; Hayes, 2009), these findings indicated that self-perceived mate value partially mediated the relation between heterosexual rejection and absent participants' mating behavior tendencies.

### Discussion

In this study, we found that one's self-perceived mate value was lowered by heterosexual rejection while raised indistinctively by heterosexual acceptance, which indicated that rejection elicited a more apparent change in self-perceived mate value than acceptance. However, self-perceived mate value of males was significantly higher than the control group while females was not in the situation of acceptance. Males' mating behaviors are mostly related to their self-perceived mate value. Males of high self-perceived mate value have more opportunities to get in touch with the opposite sex to gain short-term relationships (Surbey and Brice, 2007), while females' mating behaviors are partially related to their surroundings (Gangestad and Simpson, 2000) and it's harder for females to mate with their age increases, thus they will lower their criteria to actively mate.

Mate selection is a complex process, during which individuals need to choose their ideal companion from the opposite sex. This requires them to set a desired level and try their best to approach the actual situation. Only in this way can individuals ensure success in finding the right spouse in the mating market (Penke and Dennissen, 2008). Mate selection is a mutual process and it is important to set a properly desired level. One is also being judged and chosen while they are choosing others. If the desired level is set too high, individuals are likely to miss many potential spouses (Kavanagh et al., 2010). The current study showed that, when facing rejection by the opposite sex, the level of mate expectations of the rejected declines while the accepted increases. One limitation of previous studies is that researchers just compared the accepted group with the rejected group without considering the control group. Therefore, we could not judge whether heterosexual rejection lowers mate expectations or heterosexual acceptance increases mate expectations or that they coexist. Thus, we added the control group to prove that heterosexual rejection lowers mate expectations and heterosexual acceptance does not increase mate expectations significantly. With the decline of mate expectations, individuals tend to choose those who they can pursue more easily to ensure the success of mating.

Self-perceived mate value and mate expectation experienced a significant decline when heterosexual rejection occurred and a significant increase when heterosexual acceptance happened. Meanwhile, self-perceived mate value completely mediates the relationship between heterosexual rejection and mate expectation. In this sense, heterosexual rejection or acceptance influences expectations via one's self-perceived mate value. Based on this mechanism, we speculate that individuals with high mate expectations know their self-perceived mate value more accurately through heterosexual passive rejection or acceptance and can further regulate their mate expectations. In contrast, individuals with low mate expectations ensure their self-perceived mate value more precisely through heterosexual positive rejection or acceptance and raise their expectations accordingly.

There are few studies on the influence of heterosexual rejection on mating behavior tendencies. Previous studies mainly concentrated on the relationship between social rejection and individuals' behaviors. Rejected individuals are motivated to regain a sense of connection given that experiences of rejection and ostracism threaten their needs of belonging (Knowles, 2014), so it is necessary to restore when being rejected or excluded (Maner et al., 2007). However, other studies showed that rejection did not necessarily lead individuals to rebuild relationships; on the contrary, it resulted in a reduction in pro-social behaviors (Twenge et al., 2007), self-frustration (Twenge et al., 2002), and aggressive behaviors (Twenge et al., 2001).

In order to further verify whether mating sociometer plays a role in the mating behavior tendencies, we investigated the mediating role of self-perceived mate value between heterosexual rejection and mating behavior tendencies. The results showed that one's self-perceived mate value partially mediates the relationship between heterosexual rejection and mating behaviors. However, this mediation effects only exist in absent participants' mating behavior tendency. For present participants, self-perceived mate value reported no mediating effect.

## General discussion

On the basis of the sociometer theory, Kirkpatrick and Ellis (2001) put forward the specific domain sociometer, in which self-esteem was considered as a component of the sociometer since it consists of diverse subsets monitored in respective domains. From the perspective of evolutionary psychology, sexual relations are particularly important to people. In this study, we have validated and applied the specific domain sociometer in the field of mating. Furthermore, Kirkpatrick and Ellis (2001) named the specific domain sociometer in the field of mate selection the mating sociometer. Penke and Dennissen (2008) approved of this sociometer and hypothesized that there was a certain psychological mechanism existing in the field of mate selection to monitor individuals' mating behaviors.

Previous studies preliminarily verified the existence of the mating sociometer and proved that self-esteem mediated the relationship between heterosexual acceptance or rejection and mate expectation (Ruan and Zhang, 2012). However, these studies did not elaborate on the difference between overall self-esteem and specific self-esteem nor compare the rejected groups with the accepted groups to confirm whether acceptance or rejection caused the change of mate expectations. To remedy previous shortcomings, we first differentiated between self-esteem and self-perceived mate value and found that both heterosexual and same-sex rejection influenced one's self-esteem with no significant difference. What's more, self-perceived mate value of individuals in the heterosexual rejected group lowered significantly when compared with the control group. These results indicated that there was a difference between overall self-esteem and specific self-esteem: overall self-esteem has domain generality to function in all domains while the specific self-esteem only affects in a certain domain.

The current study investigated the effect of heterosexual rejection on mating behaviors in three groups, namely, the rejected group, the accepted group, the rejected group, and the control group. The results showed that the accepted group and the control group reported no significant difference in both mate expectations and mating behavior tendencies while these of the rejected group experienced a significant decline. It effectively indicated that it was heterosexual rejection not heterosexual acceptance that influenced one's mating behaviors.

In addition, we investigated the probable mediating effect of self-perceived mate value in the relationship between heterosexual rejection and mating behaviors to verify the effectiveness of the mating sociometer. We found that self-perceived mate value not only completely mediated the relationship between heterosexual rejection and mate expectation, but also mediated the relationship between heterosexual rejection and absent participants' mating behavior tendencies.

In summary, this study verified the existence of the mating sociometer and its ability to monitor individuals' heterosexual relationships. When heterosexual rejection occurs, the mating sociometer would send a signal to inform individuals of their heterosexual relationship status as well as inspire them to take some adaptive measures to protect themselves. In this case, individuals either lower their mating choice criteria or reduce their chances of getting in touch with the opposite sex.

However, he present study has several limitations. First, the participants recruited in this study are all college students and the number of the sample is too small and not representative enough. Under these circumstances, the results concluded in this study have not been promoted as good as they need to be. For future studies, it would better to expand the amount and scope of the participants in order to consummate the conclusions. Second, to ensure the ecological validity of the experiment and reduce the difficulties of manipulation, strict content and quality control of communication should be applied in the speed-dating activity. However, after being tested under different types of manipulations, individuals' psychological and behavioral reactions were not only affected by experimental treatment, but also other uncontrollable factors during the activity, which thereby produced an interaction with experimental feedbacks. For further research, some standardized manipulations should be taken on the contents of communication. Third, only an explicit measurement to investigate participants' mating choices was conducted. However, evading or approaching the opposite sex belongs to the category of mate choices that individuals need some time to ponder carefully. The experiment will be more scientific if some implicit measurements are put into practice. Fourth, we did not record participants' current relationship status which maybe a potential confound. Further research might as well consider participants' current relationship status as a variable. Fifth, only female participants were selected to test the effectiveness of the three scene-priming materials in Experiment 1. We expect that it would be better to select both genders to evaluate materials.

### Conflict of interest statement

The authors declare that the research was conducted in the absence of any commercial or financial relationships that could be construed as a potential conflict of interest.
