# Supplementary material for: Heterosexual Rejection and Mate Choice: A Sociometer Perspective
Source: Front Psychol. 2015 Dec 1;6:1846. doi: 10.3389/fpsyg.2015.01846 (PMC4665087; doi:10.3389/fpsyg.2015.01846)
Supplement: Supplementary file 1 [file DataSheet1.DOCX]

**Appendix**

Three scenarios used in Experiment 1

The heterosexual rejection group:

In a big party, both males and females could invite their favorite heterosexual partners to dance with them. When you invited a heterosexual partner, you were rejected. Please answer the following: (a) What did you think when being rejected? (b) Please think carefully whether you experienced similar rejection by the opposite sex and recall it as vividly as possible. (c) Describe the process of being rejected, your feelings in that moment, and subsequent influences from being rejected.

The same-sex rejection group:

In a three-legged race, all participants were allowed to compete and one should invite a partner to form a group. When you invited a same-sex partner nearby, you were rejected. Please answer the following: (a) What did you think when being rejected? (b) Please think carefully whether you experienced similar rejection by the same-sex and recall it as vividly as possible. (c) Describe the process of being rejected, your feelings in that moment, and subsequent influences from being rejected.

The heterosexual acceptance group:

In a three-legged race, all participants were allowed to compete and one should invite a partner to form a group. When you invited a same-sex partner nearby, you were accepted. Please answer the following: (a) What did you think when being accepted? (b) Please think carefully whether you experienced similar acceptance and recall it as vividly as possible. (c) Describe the process of being accepted, your feelings in that moment, and subsequent influences from being accepted.

The instructions in each condition were all “Please read the following carefully and answer the following questions as vividly as possible”.
